# Prevalence and determinants of anemia due to micronutrient deficiencies among children aged 12–59 months in India–Evidence from Comprehensive National Nutrition Survey, 2016–18

**DOI:** 10.1371/journal.pgph.0002095

**Published:** 2024-01-18

**Authors:** Kapil Yadav, Gomathi Ramaswamy, Surabhi Puri, Kashish Vohra, Thejas Achary, Abhishek Jaiswal, Ravneet Kaur, Mohan Bairwa, Archana Singh, Vani Sethi

**Affiliations:** 1 Centre for Community Medicine, All India Institute of Medical Sciences, New Delhi, India; 2 National Centre of Excellence and Advanced Research on Anemia Control, All India Institute of Medical Sciences, New Delhi, India; 3 Department of Community Medicine and Family Medicine, All India Institute of Medical Sciences, Bibinagar, India; 4 Employee State Insurance Corporation Medical College and Hospital, Faridabad, Haryana, India; 5 Department of Biochemistry, All India Institute of Medical Sciences, New Delhi, India; 6 United Nations International Children’s Emergency Fund, Regional Office for South Asia, Kathmandu, Nepal; KEM Hospital Pune, INDIA

## Abstract

The present study aimed to estimate the prevalence of anemia, and anemia with micronutrient deficiencies (iron/ vitamin B12/ folic acid) and their determinants among children aged 12–59 months in India. Comprehensive National Nutritional Survey (2016–2018) is Asia’s largest nutrition survey conducted among 0–19 years aged children in India. We used generalised linear model (modified Poisson) with adjusted prevalence ratio (aPR) to assess the socio-economic and biochemical factors associated with anemia and anemia with micronutrient deficiencies amongst children aged 12 to 59 months. Out of the total of 11,237 children included in the study, 40.5% (95%CI:38·6–42·6) were anemic, 30.0% (95%CI:27·8–32·4) had anemia with micronutrient deficiencies and 60.9% (95%CI:58·2–63·5) had micronutrient deficiencies with or without anemia. Younger age (aPR(95%CI) for one year old: 1.9(1.5–2.4), two year old: 1.8(1.5–2.2), three year old: 1.4(1.2–1.7) compared to four year old children) and lower educational status of the mother (mothers without formal schooling aPR(95%CI):1.4(1.1–1.8); 1–9 standards: 1.4(1.2–1.7)) vs mother educated with high school and above, consumption of less than 100 iron-folic acid tablets during pregnancy (aPR(95%CI):1.3(1.0–1.7) vs consumption of ≥ 180 tablets, any self-reported illness among children within two weeks preceding the interview (aPR(95%CI):1.2(1.1–1.4) vs no-illnesses, iron deficiency (aPR(95%CI):2.2(2.0–2.6)) and zinc deficiency (aPR(95%CI):1.3(1.1–1.4)) were associated with anemia in children. Among anemic, the children from scheduled tribe (aPR(95%CI):1.4(1.1–1.8)) vs other caste categories, and those following unsafe child faeces disposal practices (aPR(95%CI):1.2(1.0–1.4)) vs those who follow safe faeces disposal practices had higher chance of having micronutrient deficiency. One third of children aged 12–59 months had anemia with micronutrient deficiency (iron/ folic acid/ vitamin B12). More than half of children had micronutrient deficiencies irrespective of anemia. Micronutrient deficiencies, antenatal IFA intake, safe hygiene practices need to be strengthened to leave no stone unturned in control of anemia among under-five children in India.

## Introduction

Globally, anemia is a major public health concern affecting 1.62 billion population [1]. The Global Burden of Disease 2017 estimates that around 45% of population in India are anemic [2]. National Family Health Survey 5 (NFHS 5: 2019–21) of India reported high burden of anemia in under-five age group with an estimated prevalence of 67.1% [3]. Based on the NFHS 5 data anemia is considered as a severe public health problem among under-five children in India as World Health Organization (WHO) classifies anemia as severe public health problem when the prevalence of anemia is more than 40% in the country [4, 5].

WHO classifies hemoglobin value of less than 11 g/dl as cut-off for diagnosis of anemia among children less than five years [6]. Anemia during formative years can result in poor cognitive development, growth delay, and increased susceptibility to infections [5, 7]. More than one condition might be associated with anemia leading to its multifactorial causation [8]. Anemia can be broadly categorized as nutritional anemia and non-nutritional anemia based on causation [8] Micronutrients such as iron, vitamin B12, folic acid, vitamin A, and zinc play a major role in hemoglobin metabolism, and deficiency of any of these can result in nutritional anemia. Non-nutritional anemia could be due to genetic hemoglobin disorders, infections, inflammations and environmental conditions. Under-five children have high nutritional requirements for iron and other micronutrients, making them prone for developing nutritional anemia.

Iron deficiency is one of the most common causes of anemia and 50% of anemia reported is amenable to iron supplementation [8–10]. However, iron deficiency might be associated with only one-fourth (25%) of total anemia among preschool children [11]. There is limited information on the proportion of anemia due to iron deficiency especially among children under five years of age. Also, there is not much information available on the magnitude of other micronutrient deficiencies associated with anemia amongst children [9]. India launched Anemia Mukt Bharat (AMB) program in 2018, with a target of three percent reduction in the burden of anemia every year [12]. The program planned to address non-nutritional causes of anemia along with continued efforts to tackle nutritional anemia. Iron and folic acid (IFA) supplementation is the main stay of anemia control strategy among under-five children under AMB. However, it is important to understand the determinants of anemia especially other micronutrient deficiencies in under-five children in order to bring down the prevalence of anemia with targeted interventions.

The Comprehensive National Nutrition Survey (CNNS, 2018) is the largest nutrition survey conducted in India among individuals aged 0–19 years which has the data on micronutrient and macronutrient status among the surveyed population. CNNS provides an opportunity to understand the micronutrient deficiencies and various factors associated with anemia [13]. The present study was undertaken to estimate the prevalence of 1) anemia, 2) anemia with micronutrient deficiencies (iron/ vitamin B12/ folic acid) and 3) the determinants of the above outcomes amongst 12 to 59 months aged children.

## Material and methods

The CNNS survey data was collected from 24 February 2016 to 26 October 2018 among the 112,316 children and adolescents aged 0 to 19 years from all the 30 states of India [13]. The survey adopted a multi-stage, stratified, probability proportional to size (PPS) cluster sampling design. The household and individual questionnaires for under-five children were answered by parents or caregivers. Children with a physical deformity, ongoing illness, or chronic illness were excluded from the survey. All sampled children aged 12–59 months were contacted for survey and biological sample collection.

Trained phlebotomists collected eight millilitres (ml) of a blood sample from children aged 12 months to 59 months. The vacutainer tubes with blood samples were transported in cold boxes to the pre-identified nearest sample collection center for laboratory analysis. Hemoglobin was estimated from whole blood samples using a five-part automated cell counter (Beckman colter)/ Photometric estimation (cyanmethemoglobin method). Serum ferritin was estimated using the immunoassay technique, while erythrocyte folate and vitamin B 12 levels were estimated in the competitive immunoassay using direct chemiluminescence (Siemens Centaur). For the present analysis, we included the data of children aged 12 to 59 months with valid values for hemoglobin, s. ferritin, vitamin B12 and folate.

The primary outcomes for this study were a) anemia (altitude adjusted hemoglobin <11g/dl) and b) anemia with micronutrient deficiency—altitude adjusted hemoglobin <11g/dl with at least one of iron (serum ferritin <12 μg/l) or vitamin B12 (serum vitamin B12 < 203 pg/ml) or folate (serum erythrocyte folate < 151 ng/ml) deficiency [8, 14–17]. We considered serum vitamin A, Zinc and vitamin D covariates to examine their association with anemia and also anemia with micronutrient deficiencies.

We used a conceptual framework with the basic or underlying and immediate determinants of anemia based on the existing literature [18]. A total of 31 covariates (socio-economic factors, maternal factors including iron and folic acid intake during pregnancy, sanitation and hygiene, infant and young child feeding practices, anthropometric parameters, and biological parameters including micronutrient conditions such as iron, folate, vitamin B12, vitamin A and zinc, and CRP) were identified based on the conceptual framework and were included for assessing the determinants of the anemia among 12 to 59 months children.

We categorized quantitative variables such as mothers age, mothers’ education and birth spacing based on guidelines and existing literature for regression analysis. IFA intake was categorized into four categories—never consumed, <100, 100–179, and ≥180 IFA tablets consumed [12, 19]. Dietary diversity was categorized as per Food and Agricultural Organization (FAO) recommendations [20]. Sanitation and hygiene practices were categorized based on WHO and NFHS guidelines [21]. Similarly, we followed standard cut-offs for biological parameters [22, 23].

Socio-economic status was calculated using wealth index which includes indicators related to household’s assets and resources such as ownership of televisions, bicycles, materials used in house, availability of water and sanitation, and variety and quantity of consumer goods owned. To determine the wealth quintiles, scores were assigned to households based on a weighted principal components analysis adjusted at national and state level [24]. The data analysis was conducted using Stata version 14.0 (StataCorp LLC, College Station, Texas). The proportion of anemia and anemia with micronutrient deficiencies among children 12–59 months of age were expressed as percentages with 95% confidence interval (CI). We used the generalised linear model (modified Poisson) for analysis to assess the factors associated with the above outcomes and the prevalence ratio with 95% CI were calculated as a measure of association. We considered independent variables with a p-value less than 0.25 in bivariate analysis for inclusion in the multivariable regression. We checked the multicollinearity and factors with more than five collinearity score were excluded in the final multivariable model. We considered the Akaike Information Criterion (AIC) and Bayesian Information Criterion (BIC) values for assessing the model fitness and finalized the model, which had the lowest AIC and BIC values. We generated maps in QGIS software to show the survey weight-adjusted prevalence of anemia and anemia with micronutrient deficiencies across states.

### Ethics approval

The ethics approval for the CNNS was obtained from the Population Council’s Institutional Review Board (IRB), New York, USA and PGIMER, Chandigarh, India, before initiating the survey. Informed consent (signed/ thumb impression from illiterate mothers/ caregivers) was obtained from mothers/ caregivers of the under-five children in presence of a witness in their local language. Ethics approval was not obtained again specific for this study.

## Results

Of the 17230 children aged 12 to 59 months included in the CNNS survey, 11237 (65.2%) children had a valid hemoglobin value. Of these 11237, 52% were males. Around 75% of the children were from rural area and 16% belonged to poorest wealth quintile. Thirty five percent of children were underweight (<2SD) and 47% had reported some illness in last two weeks preceding the survey. Around 60% of the mothers were in the age group of 25–34 years. One third of the mothers never attended school and almost 75% of the mothers were unemployed during the time of survey. Twenty four percent of the mothers reported that they did not consume IFA during pregnancy and 41% consumed less than 100 IFA tablets during pregnancy. More than 50% of the mothers were disposing their child faeces in unsafe manner and did not follow adequate hand sanitation practices (Table 1).

**Table 1:** Characteristics of children aged 12–59 months in India from Comprehensive National Nutrition Survey with anemia and anemia with micronutrient deficiencies, 2016–2018.

Characteristics	Total		Anemia		Total		Anemia with micronutrient deficiencies	
	N^a^^,^^b^	(%)^e^	n^b^	(%)^d^	N^b^^,^ ^c^	(%)^e^	n^b^	(%)^d^
**Total**	11237	(100)	4557	(40.5)	3635	(100)	2639	(72.6)
**Socio economic factors**								
**Age (in years)**	n = 11233				n = 3629			
1	1704	(15.2)	1065	(62.5)	852	(23.5)	646	(75.7)
2	2587	(23.0)	1318	(51.1)	1009	(27.8)	740	(73.3)
3	3291	(29.3)	1239	(37.6)	1031	(28.4)	764	(74.1)
4	3651	(32.5)	930	(25.4)	737	(20.3)	485	(65.7)
**Sex**	n = 11232				n = 3630			
Male	5851	(52.1)	2374	(40.6)	1947	(53.6)	1377	(70.8)
Female	5381	(47.9)	2179	(40.5)	1683	(46.4)	1257	(74.6)
**Area**	n = 11237				n = 3635			
Urban	2848	(25.3)	1062	(37.3)	890	(24.5)	730	(82.1)
Rural	8389	(74.7)	3495	(41.6)	2745	(75.5)	1909	(69.5)
**Religion**	n = 11233				n = 3619			
Hindu	9032	(80.4)	3757	(41.6)	2976	(82.2)	2190	(73.6)
Muslim	1646	(14.6)	567	(34.4)	446	(12.3)	300	(65.9)
Christian	305	(2.7)	101	(33.1)	84	(2.3)	53	(63.6)
Sikh	125	(1.1)	48	(38.4)	42	(1.2)	39	(93.6)
Others	125	(1.1)	81	(64.8)	71	(2.0)	51	(71.6)
**Caste**	n = 10515				n = 3417			
SC	2532	(22.5)	1100	(43.4)	858	(25.1)	628	(73.2)
ST	1423	(12.7)	755	(53.1)	627	(18.3)	496	(79.1)
OBC	4663	(41.5)	1708	(36.6)	1323	(38.7)	965	(73.0)
Others	1897	(16.9)	736	(38.8)	609	(17.8)	406	(66.6)
**Wealth Index**	n = 11233				n = 3630			
Poorest	1751	(15.6)	815	(46.6)	609	(16.8)	344	(56.5)
Poor	2360	(21.0)	1069	(45.3)	872	(24.0)	594	(68.1)
Middle	2496	(22.2)	989	(39.6)	814	(22.4)	580	(71.2)
Rich	2376	(21.1)	905	(38.1)	701	(19.3)	579	(82.5)
Richest	2250	(20.0)	774	(34.4)	634	(17.5)	538	(84.9)
**Maternal factors**								
**Mother’s age (in years)**	n = 11113				n = 3635			
≤24	3242	(29.2)	1426	(44.0)	1147	(31.9)	828	(72.2)
25–34	6846	(61.6)	2717	(40.0)	2189	(60.8)	1598	(73.0)
≥35	1025	(9.2)	367	(35.8)	264	(7.3)	187	(70.8)
**Mother’s education**	n = 11226				n = 3630			
Never attended school	3134	(27.9)	1388	(44.3)	1104	(30.4)	720	(65.2)
1–9 standard	4459	(39.7)	1883	(42.2)	1515	(41.7)	1122	(74.0)
High School and above	3633	(32.4)	1279	(35.2)	1011	(27.9)	792	(78.4)
**Mother’s employment**	n = 11204				n = 3625			
Not gainfully employed	8213	(73.3)	3270	(39.8)	2586	(71.3)	1871	(72.4)
Employed	2991	(26.7)	1274	(42.6)	1039	(28.7)	759	(73.1)
**Birth spacing (in years)**	n = 6844				n = 2394			
<3	3708	(54.2)	1644	(44.3)	1350	(56.4)	974	(72.1)
≥ 3	3136	(45.8)	1272	(40.6)	1044	(43.6)	760	(72.8)
**IFA intake in pregnancy**	n = 10622				n = 3635			
Not consumed	2661	(25.1)	1114	(41.9)	838	(24.2)	568	(67.8)
<100 tablets	4670	(44.0)	2012	(43.1)	1677	(48.4)	1189	(70.8)
100–179 tablets	2034	(19.1)	773	(38.0)	611	(17.6)	474	(77.5)
≥180 tablets	1257	(11.8)	420	(33.4)	341	(9.8)	274	(80.2)
**IYCF and dietary supplements**								
**Exclusive Breastfeeding** n = 10508					n = 3446			
< 6 months	2708	(25.8)	1075	(39.7)	830	(24.1)	602	(72.5)
≥ 6 months	7800	(74.2)	3209	(41.1)	2616	(75.9)	1916	(73.2)
**Dietary diversity**	n = 11172				n = 3614			
Low (< 4 food groups)	7235	(64.8)	3113	(43.0)	2490	(68.9)	1859	(74.6)
Adequate (≥ 4 food groups)	3937	(35.2)	1421	(36.1)	1124	(31.1)	765	(68.0)
**Received IFA in last 1 week** n = 11142					n = 3601			(
No	10322	(92.6)	4189	(40.6)	3333	(92.6)	2438	(73.1)
Yes	820	(7.4)	332	(40.4)	268	(7.4)	169	(62.8)
**Vitamin A received in last 6 months** n = 10430					n = 3635			
No	7180	(68.8)	2805	(39.1)	2189	(65.0)	1607	(73.4)
Yes	3250	(31.2)	1380	(42.4)	1178	(35.0)	853	(72.4)
**Deworming dose received in last 6 months** n = 11077					n = 3575			
No	7070	(63.8)	3010	(42.6)	2378	(66.5)	1775	(74.6)
Yes	4007	(36.2)	1468	(36.6)	1197	(33.5)	828	(69.2)
**Sanitation and hygiene**								
**Child feces disposal practices** n = 11232					n = 3630			(
Safe	4792	(42.7)	1720	(35.9)	1381	(38.0)	1046	(75.7)
Unsafe	6440	(57.3)	2833	(44.1)	2249	(62.0)	1588	(70.6)
**Handwashing practices**	n = 11237				n = 3635			
Adequate	4424	(39.4)	1819	(41.1)	1477	(40.7)	1106	(74.9)
Inadequate	6813	(60.6)	2739	(40.2)	2148	(59.3)	1533	(71.0)
**Main drinking water source** n = 11150					n = 3606		(	
Unimproved	867	(7.8)	407	(46.9)	325	(9.0)	254	(78.2)
Improved	10283	(92.2)	4116	(40.0)	3281	(91.0)	2369	(72.2)
**Anthropometric parameters and health status**								
**Self-reported illness in last 2 weeks** n = 10508					n = 3631			
No	5922	(52.8)	2161	(36.5)	1734	(47.8)	1321	(76.2)
Yes	5304	(47.2)	2388	(45.0)	1897	(52.2)	1314	(69.3)
**Chronic disease**	n = 11232				n = 3631			
No	10874	(96.8)	4444	(40.0)	3543	(97.6)	2577	(72.7)
Yes	357	(3.2)	109	(30.4)	88	(2.4)	58	(66.0)
**Stunted**	n = 10914				n = 3635			
No	6960	(63.8)	2398	(34.4)	1939	(55.1)	1425	(71.7)
Yes	3954	(36.2)	1996	(50.5)	1580	(44.9)	1133	(73.5)
**Wasted**	n = 10795				n = 3694			
No	9129	(84.6)	3646	(39.9)	2917	(83.5)	2184	(74.9)
Yes	1666	(15.4)	725	(43.5)	577	(16.5)	360	(62.3)
**Underweight**	n = 10983				n = 3530			
No	7071	(64.4)	2565	(36.3)	2047	(58.0)	1539	(69.0)
Yes	3912	(35.6)	1880	(48.0)	1483	(42.0)	1023	(75.2)
**Biochemical parameters**								
**Iron deficiency**	n = 8859							
No	6190	(69.9)	1806	(29.2)	-	-	-	-
Yes	2669	(30.1)	1785	(66.9)	-	-	-	-
**Folate deficiency**	n = 10394							
No	7966	(76.6)	3332	(41.8)	-	-	-	-
Yes	2428	(23.4)	899	(37.0)	-	-	-	-
**Vitamin B12 deficiency**	n = 8279							
No	7123	(86.0)	2843	(39.9)	-	-	-	-
Yes	1156	(14.0)	566	(48.9)	-	-	-	-
**Vitamin A deficiency**	n = 7427				n = 2652			
No	6068	(81.7)	2382	(39.3)	2108	(79.5)	1517	(71.9)
Yes	1359	(18.3)	672	(49.4)	544	(20.5)	346	(63.6)
**Zinc Deficiency**	n = 8002				n = 2693			
No	6474	(80.9)	2497	(38.6)	2131	(79.1)	1544	(72.4)
Yes	1528	(19.1)	667	(43.6)	562	(20.9)	355	(63.1)
**Vitamin D deficiency**	n = 8938				n = 3424			
No	7681	(85.9)	3117	(40.6)	2912	(85.0)	2062	(70.1)
Yes	1257	(14.1)	541	(43.0)	512	(15.0)	372	(72.6)
**CRP**	n = 8767				n = 3042			
Normal CRP	7896	(90.1)	3081	(39.0)	2671	(87.8)	1970	(73.7)
High CRP	871	(9.9)	441	(50.6)	371	(12.2)	180	(48.7)

Footnote

^a^Number of children with hemoglobin values available

^b^Weighted count

^c^Number of children with hemoglobin <11g/dl with valid data for iron, Vitamin B12 and folate levels

^d^Row percentage in the given category

(Definitions: *Anemia*—Hemoglobin <11g/dl, *Anemia with micronutrient deficiencies*: Hemoglobin <11g/dl with iron or VitaminB12 or folate deficiencies; *Iron deficiency*: serum ferritin <12 ng/ml (when CRP≤5ng/ml) or serum transferring receptor level -sTfR ≥1·76 mg/L and sTfR-F index ≥ 1·63) L (when CRP >5ng/ml), *folate deficiency*: erythrocyte folate: <151 ng/mL, *Vitamin B12 deficiency*: serum cyanocobalamin <203 pg/mL)

Out of 11237 children aged 12–59 months, anemia was present in 4557 (40.5%; 95% CI: 38·6–42·6) children. The prevalence of anemia was almost similar among the boys and girls. Among the 4557 anemic children, 3635 children had valid hemoglobin, serum ferritin, CRP, erythrocyte folate, and vitamin B12 values, of which 2639 (72.6%, 95% CI: 68·7–76·1) had anemia with micronutrient deficiencies (at least one of iron, vitamin B12, folate) (Table 1).

**Fig 1:**
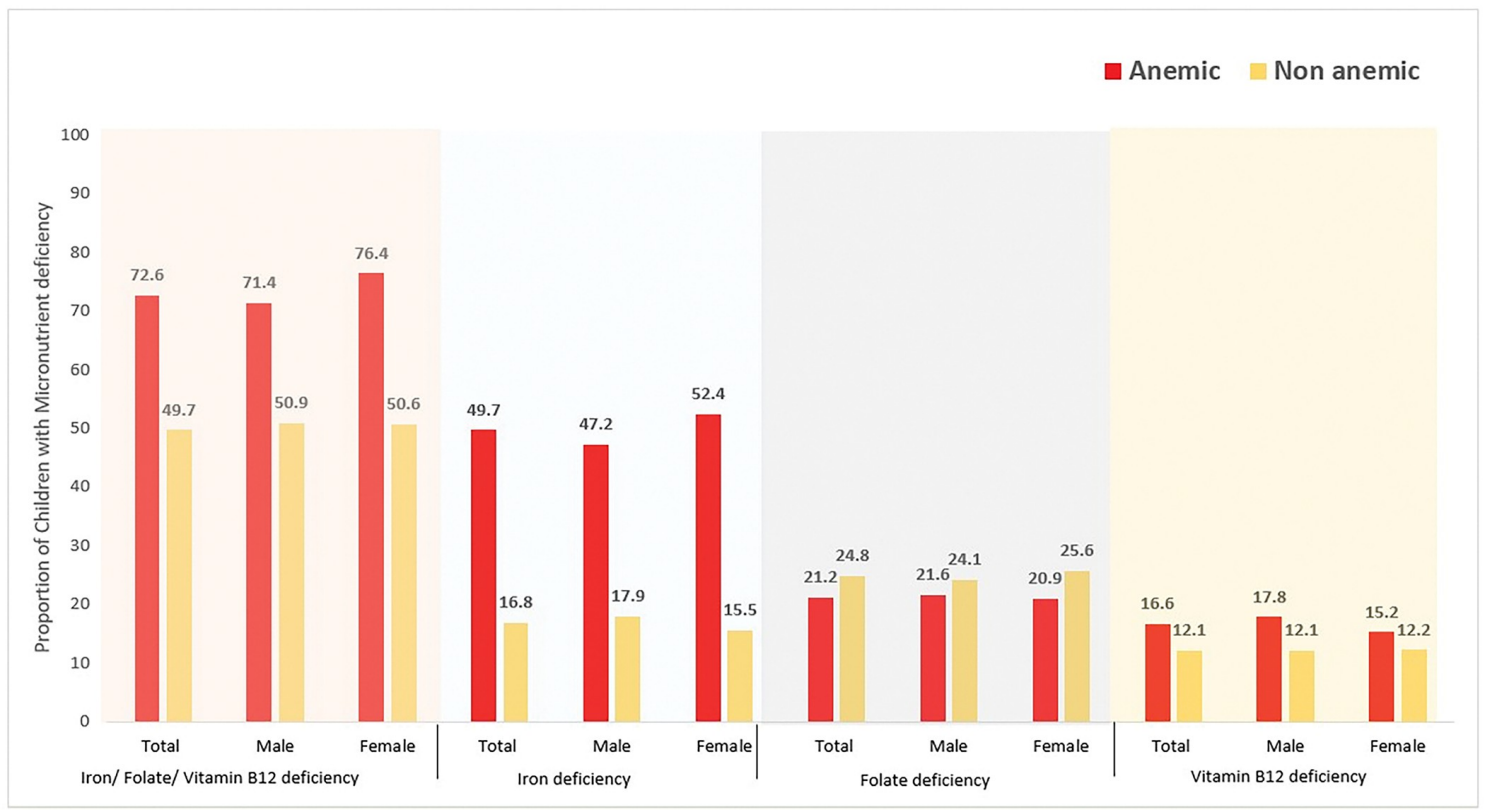
Prevalence Iron, Folate and Vitamin B12 deficiency based on gender and anemia status among the children aged 12 to 59 months in India from Comprehensive National Nutritional Survey, 2016–2018. [Note: Iron deficiency: serum ferritin <12 ng/mL (when CRP≤5ng/ml) or serum transferring receptor level -sTfR ≥1·76 mg/L and sTfR-F index ≥ 1·63) (when CRP >5ng/ml), folate deficiency: erythrocyte folate: <151 ng/mL, Vitamin B12 deficiency: serum cyanocobalamin <203 pg/mL].

A high proportion of micronutrient deficiency (overall prevalence: 60.9%, 95%CI: 58.2–63.5) was observed among anemic children (72.6%, 95%CI: 68.7–76.1) than non-anemic children (49.7%, 95%CI: 46.0–53.3). (Fig 1) In case of individual micronutrient deficiencies, iron deficiency (overall prevalence: 30.4%, 95%CI: 28.3–32.6) was more prevalent among children with anemia (49.7%, 95%CI: 46.1–53.3) than children without anemia (16.8%, 95% CI: 14.8–18.9). However, we did not observe much difference in the proportion of vitamin B12 deficiency (overall prevalence: 13.8%, 95% CI:1.7–16.2; among anemic children: 16.6%, 95% CI:12.8–21.3; among non-anemic children: 12.1%, 95%CI: 10.1–14.5) and folate deficiency (overall prevalence: 23.4%, 95%CI: 21.2–25.7; among anemic children: 21.1%, 95%CI: 18.1–24.7 among non-anemic children: 24.8%, 95%CI: 22.4–27.4) based on the presence of anemia.

**Fig 2:**
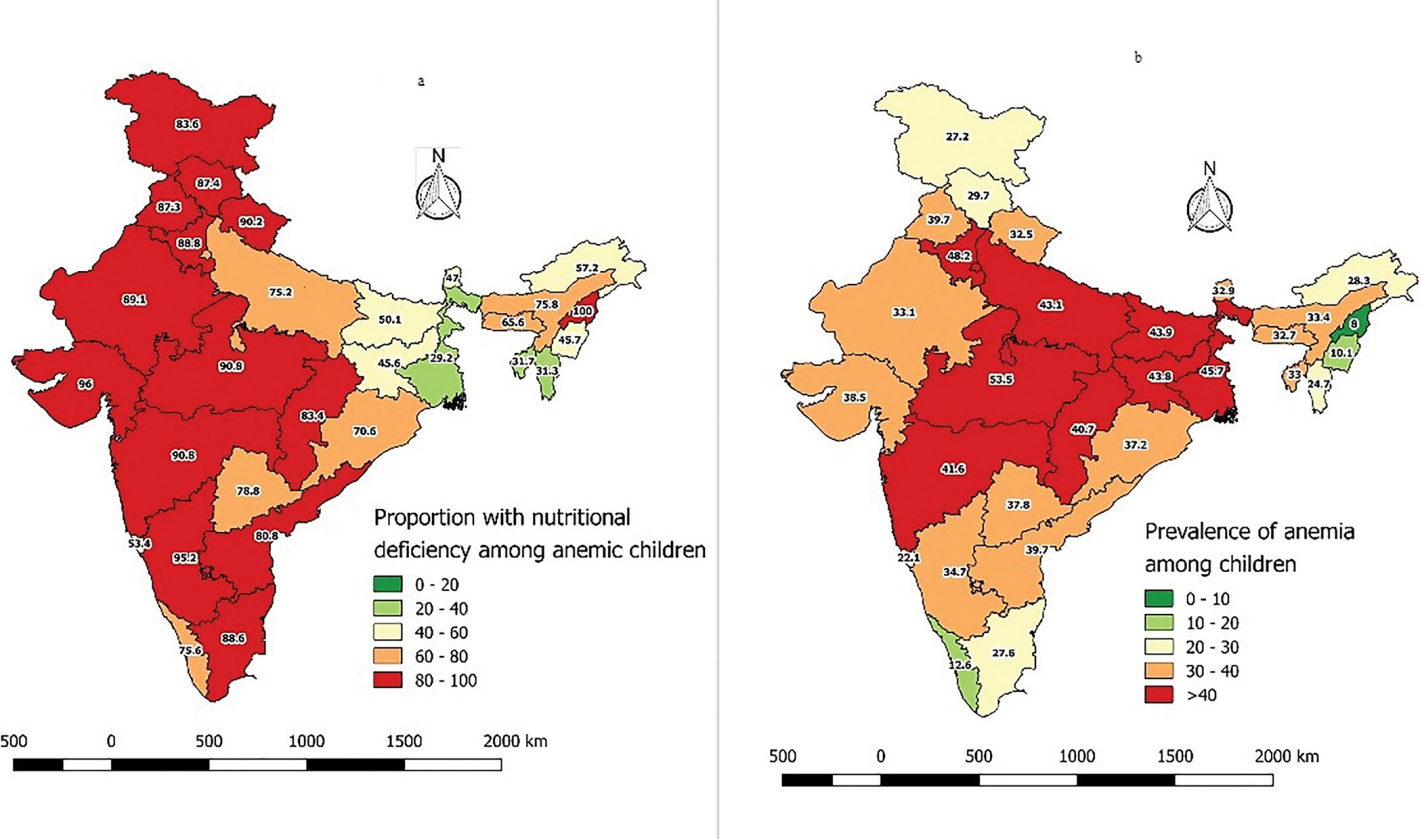
Prevalence of anemia (a) and anemia with micronutrient deficiencies (b) among children aged 12–59 months across 30 states in India from Comprehensive National Nutritional Survey, 2016–2018. [Note: Anemia—Hemoglobin <11g/dl. Anemia with micronutrient deficiencies: Hemoglobin <11g/dl with iron or VitaminB12 or folate deficiencies. (Iron deficiency: serum ferritin <12 ng/mL (when CRP≤5ng/ml) or serum transferring receptor level -sTfR ≥1·76 mg/L and sTfR-F index ≥ 1·63) (when CRP >5ng/ml), folate deficiency: erythrocyte folate: <151 ng/mL, Vitamin B12 deficiency: serum cyanocobalamin <203 pg/mL). Source for India admin shape files is https://diva-gis.org/download which is free and open source. Software used to create Fig 2, QGIS (previously known as Quantum GIS), is a free and open source cross platform desktop geographic information system (GIS) application that supports viewing, editing and analysis of geospatial data].

The state wise prevalence of anemia and anemia with micronutrient deficiency is depicted in Fig 2. Since the data collection for CNNS was done in 2016–2018 the analysis for the states formed after 2016 was not done separately (Jammu and Kashmir was considered as a single state). The data form 30 states (29 states and 1 union territory) indicates that 40% of under-five children have anemia in eight states. However, 20 states (67%) reported that more than 60% children with anemia have micronutrient deficiency in India. Among the eight states with anemia burden more than 40%, four states had micronutrient deficiency more than 80% among the anemic children. One state had 75% micronutrient deficiency among anemic children and two had 45–50% micronutrient deficiency among anemic children. Only one state had 30% micronutrient deficiency among anemic children.

Children less than 4 years of age (aPR(95%CI) for children aged one-year: 1.9 (1.5–2.4), two-year: 1.8 (1.5–2.2), three-year: 1.4 (1.2–1.7)); mothers with no or low education level (aPR(95%CI) for no schooling: 1.4 (1.1–1.8); 1–9 standard: 1.4 (1.2–1.7)); consumption of less than 100 IFA tablets during the pregnancy (aPR (95% CI): 1.3 (1.0–1.7); and children with self-reported illness in the last two weeks (aPR(95% CI): 1.2 (1.1–1.4) had higher risk of having anemia. Children with iron (aPR(95% CI): 2.2 (2.0–2.6)) and zinc deficiencies (aPR(95% CI): 1.3 (1.1–1.4)) were more likely to have anemia. Other micronutrient deficiencies such as vitamin B12, folate, vitamin A, and vitamin D were not statistically associated with anemia (Table 2).

**Table 2:** Factors associated with anemia and anemia with micronutrient deficiencies among children aged 12–59 months in India from Comprehensive National Nutrition Survey, 2016–2018.

Characteristics	Anemia	Anemia with micronutrient deficiencies
	Adjusted PR	Adjusted PR
**Socio economic factors**		
**Age (in years)**		
1	1.9 (1.5–2.4) ^c^	1.1 (0.9–1.4)
2	1.8 (1.5–2.2) ^c^	1.1 (0.9–1.3)
3	1.4 (1.2–1.7) ^b^	1.1 (0.9–1.4)
4	1	
**Sex**		
Male	-	1
Female	-	1.0 (0.9–1.2)
**Area**		
Urban	1	1
Rural	1.0 (0.8–1.2)	0.9 (0.8–1.1)
**Religion**		
Hindu	1.0 (0.8–1.2)	1.2 (0.9–1.4)
Muslim	1	1
Christian	1.2 (0.7–1.8)	1.1 (0.7–1.7)
Sikh	0.8 (0.4–1.4)	1.6 (1.0–2.5)
Others	1.4 (0.6–3.2)	0.8 (0.4–1.5)
**Caste**		
SC	0.8 (0.6–1.0)	1.1 (0.9–1.3)
ST	0.9 (0.7–1.2)	1.4 (1.1–1.8) ^b^
OBC	0.8 (0.7–1.0) ^b^	1.1 (0.9–1.3)
Others	1	1
**Wealth Index**		
Poorest	1.2 (0.9–1.6)	0.5 (0.4–0.7) ^c^
Poor	1.0 (0.7–1.3)	0.7 (0.5–0.8) ^b^
Middle	0.9 (0.7–1.2)	0.7 (0.6–0.9) ^a^
Rich	0.9 (0.7–1.1)	0.9 (0.8–1.1)
Richest	1	1
**Maternal factors**		
**Mother’s age (in years)**		
≤24	1.0 (0.8–1.2)	-
25–34	1	-
≥35	0.9 (0.7–1.1)	-
**Mother’s education**		
Never attended school	1.4 (1.1–1.8) ^b^	0.9 (0.8–1.3)
1–9 standard	1.4 (1.2–1.7) ^c^	1.0 (0.8–1.2)
High School and above	1	1
**Mother’s employment**		
Not employed	1	-
Employed	1.0 (0.9–1.2)	-
**Birth spacing (in years)**		
<3	1.0 (0.9–1.1)	-
≥ 3	1	-
**IFA intake during pregnancy**		
Not consumed	1.2 (0.9–1.5)	1.0 (0.8–1.3)
<100 tablets	1.3 (1.0–1.7) ^b^	1.0 (0.8–1.2)
100–179 tablets	1.1 (0.8–1.5)	1.0 (0.8–1.3)
≥180 tablets	1	1
**IYCF and dietary supplements**		
**Exclusive Breastfeeding**		
< 6 months		-
≥6 months		-
**Dietary diversity**		
Low (< 4 food groups)	1.1 (0.9–1.2)	1.1 (1.0–1.2)
Adequate (≥ 4 food groups)	1	1
**Received IFA in last one week**		
No		-
Yes		¬
**Vitamin A received in last 6 months**		
No	0.9 (0.8–1.0)	-
Yes	1	-
**Deworming dose received in last 6 months**		
No	1.0 (0.9–1.2)	1.1 (0.9–1.3)
Yes	1	1
**Sanitation and hygiene**		
**Child feces disposal practices**		
Safe	1	1
Unsafe	1.1 (0.9–1.2)	1.2 (1.0–1.4) ^a^
**Main drinking water source**		
Unimproved	1.1 (0.8–1.5)	-
Improved	1	-
**Anthropometric parameters and health status**		
**Self-reported illness in last 2 weeks**		
No	1	0.9 (0.8–1.0)
Yes	1.2 (1.1–1.4) ^b^	1
**Chronic disease**		
No	1	-
Yes	0.9 (0.6–1.3)	¬-
**Stunted**		
No	1	-
Yes	1.1 (1.0–1.3)	-
**Wasted**		
No	1	1
Yes	1.0 (0.9–1.3)	0.9 (0.7–1.0)
**Underweight**		
No	1	1
Yes	1.2 (1.0–1.4)	1.1 (0.9–1.2)
**Biochemical parameters**		
**Iron deficiency**		
No	1	
Yes	2.2 (2.0–2.6) ^c^	
**Folate deficiency**		
No	1	-
Yes	1.0 (0.9–1.2)	-
**Vitamin B12 deficiency**		
No	1	
Yes	1.0 (0.8–1.2)	
**Vitamin A deficiency**		
No	1	1
Yes	1.1 (0.9–1.3)	1.0 (0.9–1.2)
**Zinc Deficiency**		
No	1	1
Yes	1.3 (1.1–1.4)^b^	1.0 (0.8–1.1)
**Vitamin D deficiency**		
No	-	-
Yes	-	-
**CRP**		
Normal CRP	1	1
High CRP	1.2 (0.9–1.4)	0.8 (0.6–0.9)^a^

Footnote

^a^p< 0.05

^b^p<0.010

^c^p<0.001.

Adjusted PR–adjusted prevalence ratio

(Definitions: *Anemia*—Hemoglobin <11g/dl, *Anemia with micronutrient deficiencies*: Hemoglobin <11g/dl with iron or VitaminB12 or folate deficiencies; *Iron deficiency*: serum ferritin <12 ng/ml (when CRP≤5ng/ml) or serum transferring receptor level -sTfR ≥1·76 mg/L and sTfR-F index ≥ 1·63) L (when CRP >5ng/ml), *folate deficiency*: erythrocyte folate: <151 ng/mL, *Vitamin B12 deficiency*: serum cyanocobalamin <203 pg/mL)

The anemia with micronutrient deficiency was relatively higher among scheduled tribe (aPR(95% CI): 1.4, (1.1–1.8)) and those following unsafe child faeces disposal practices (aPR(95% CI): 1.2, (1.0–1.4)). The risk of anemia with micronutrient deficiencies were less likely among those with high CRP levels (aPR (95% CI):0.8, (0.6–0.9)). In contrast to overall anemia, the anemia with micronutrient deficiencies was lower among the children belonging to poorest (aPR (95% CI): 0.5, (0.4–0.7)), poor (aPR(95% CI):0.7 (0.5–0.8)) and middle (aPR (95% CI):0.7 (0.6–0.9)) wealth quintiles compared to the wealthier quintile groups (Table 2).

## Discussion

Comprehensive National Nutritional Survey (2016–18) was one of the largest nationally representative nutrition surveys conducted amongst 0–19 years children in India. Of the 11237 children (12 to 59 months) included in the analysis, the burden of anemia was high with two out of five being anemic (<11 gm/dl). Among the children with anemia, almost three-fourth had micronutrient deficiencies with almost one in two having iron deficiency. Those with younger age (<4 years) and those with low mother’s education were more likely to have anemia. Children from lower wealth quintile were less likely to have anemia with micronutrient deficiencies.

The high burden of anemia among young children is a serious public health challenge in India. India has made an effort through the Prime Minister’s Overarching Scheme for Holistic Nutrition (POSHAN Abhiyaan 2018) program to improve the overall nutritional status of young children, pregnant women, and adolescent girls by creating Jan Andolan (people’s movement) for nutrition, accountability, technology integration, intersectoral coordination, and partnership [25]. Under POSHAN Abhiyaan, India revamped the anemia control program in 2018 (Anemia Mukt Bharat) to control the burden of the anemia in mission mode with a three percent annual reduction in the burden of anemia in a year [12]. However, the recently released NFHS 5 (2019–21) results showed an increase in the burden of anemia by nine percentage points compared to NFHS 5 (2019–21) in under five age group [3, 26]. Focussing on nutrition becomes more so important with multiple waves of COVID-19 in the country, which according to Global Nutrition Report (GNR) 2020 can be a ’threat multiplier for malnutrition’ [27].

There is a significant difference in the prevalence of anemia reported in NFHS 4 (56%, 2014–2015) and CNNS (41%, 2016–18). The variation in prevalence across two nationally representative surveys conducted in successive years could be due to difference in the methods used for testing hemoglobin. The NFHS 5 used capillary blood and digital hemoglobinometers for estimation of hemoglobin. Whilst, the CNNS used venous blood and autoanalyzer for estimation of hemoglobin. The nationwide prevalence surveys from other LMICs countries (using capillary blood sample and digital hemoglobinometers) such as Ethiopia (65.7%), Ghana (35.6%), Nepal (46.4%), and Pakistan (62.5%) reported slightly higher prevalence of anemia than the studies conducted using auto-analysers from the same setting [28–30]. Digital hemoglobinometers have 80% to 95% sensitivity and a similar range of specificity to diagnose anemia [31, 32]. Also, the diagnostic accuracy of the digital hemoglobinometers depends on the capillary blood sample collection procedure and sample loading techniques.

It is worrying that about three-fourth of anemia was associated with micronutrient deficiencies. This underscores the importance of tackling the micronutrient deficiencies among young children with specific nutritional interventions. WHO reports that 50% of anemia is amenable to iron supplementation [10]. Petry N et al. estimated that the proportion of anemia associated with iron deficiency might be around 25% among preschool children. The study also stated that the iron deficiency might be as low as 14% in rural areas (when anemia prevalence >40%) and 20% in high inflammation and infection countries [11]. However, in the present study 73% of anemia among the surveyed population was associated with micronutrient deficiencies such as iron or folate or vitamin B12 deficiency. This study also shows that half of the anemia was due to iron deficiency.

As per the AMB program, the proportion of children aged 6 to 59 months who received at least 8 to 10 doses of prophylactic IFA syrup (1ml—biweekly) was 6.6% in 2017–18, 8.3% in 2018–19, 14.9% in 2019–20 and 14.3% in 2020–21 and 13.6% in 2021–22 [33]. There has been some increase in IFA consumption over time, lot more efforts are required to increase IFA coverage to acceptable levels. The test and treat strategy of the AMB program may be the potential solution for identifying the children with anemia and treat them with an adequate IFA dose, while ensuring dietary diversity to meet vitamin B12 and folate requirements. The mothers who consumed less than 100 IFA tablets were more likely to have anemic children. However, the maternal anemia status and consumption of treatment doses of IFA were not captured precisely in the CNNS to provide any definite conclusion. It is essential to strengthen the IFA supply chain system considering only 30% of the women had consumed minimum 100 IFA tablets during their pregnancy and less than 15% of under-five children received the prophylactic IFA in India [34]. Also, there is need for assessing the impact of multi-micronutrient supplementation on anemia considering the high burden of folate and vitamin B12 deficiency.

The present study observed that the anemia with micronutrient deficiency were less likely to occur in children born in poorer households compared to those born in wealthier ones. Studies have reported that iron and vitamin B12 deficiency among children who were overweight and obese. However, these studies did not report the economic status of the children. Further research is required to explore the interconnection between poverty and micronutrient deficiency especially when anemia is present. We did not observe any significant association between the economic levels and the anemia status of the children. The studies conducted in African countries like Namibia and Ethiopia reported that the burden of anemia is more in poor children compared to rich children [35, 36]. The studies from India also reports similar finding that poor children are more likely to be anemic and stunted compared to their rich counter parts reinforcing the role of wealth status in macro and micro nutrient deficiency [37, 38]. A study conducted from Vietnam among the children attending primary schools also did not find any association between economic indicators and anemia. Dietary habits and non-nutritional factors might be playing major role than the economics in South Asian population. The different observations from these studies require further exploration to understand the nuances and impact of economy on anemia in Indian context. The study further confirmed that iron deficiency as one of the strong independent factors associated with anemia. However, vitamin b12 and folate did not have significant association in multivariate analysis. Interestingly zinc deficiency found to have significant association on anemia. These areas need further evidence generation to support policy making for anemia control.

We observed maternal factors played a crucial role in the nutritional status of the children. When the mother was not educated or educated less than the 10^th^ standard, the children were more likely to develop anemia, which was similar to the study from Ethiopia and NFHS 4 results from India [30, 39].

The following are the strengthen and limitations of this study. Comprehensive National Nutritional Survey (2016–18) provided one of the largest nationally representative data on anemia and micronutrient status. Hence, this study provide empirical evidence on anemia and micronutrient deficiencies. However, the survey lacks information on infections like malaria and inflammatory conditions such as fluorosis which served as important markers for non-nutritional causes for anemia. Also, almost one-third of the study population with biological samples did not have valid iron or folate, or vitamin B12 levels which restrict us from retrieving exact estimates of micronutrient deficiencies. We used modified Poisson regression over logistic regression to derive adjusted prevalence ratio (PR) instead of adjusted odds ratio (OR) which provides overestimated values when the prevalence is more than 5%.

## Conclusions

The high prevalence of anemia reported in CNNS indicates that anemia is a serious public health problem among children aged 12 to 59 months in India. Three fourth of children with anemia had micronutrient deficiencies such as iron, folate or vitamin B12 deficiency. Micronutrient deficiency and anemia are one of the major public health concerns and calls for immediate action. Given the prevalence of anemia and its association with micronutrient deficiencies, it has become a priority to control this rising burden of anemia in India. Anemia Mukt Bharat (AMB) focuses on both nutritional and non-nutritional causes of anemia by its 6x6x6 intervention strategies. Consumption of IFA during pregnancy, following safe WASH practices, control of infections management of micronutrient deficiencies are crucial to control anemia and the interventions should be implemented from infancy onwards to act on time. Hence, implementing AMB intervention strategies in all the states of India and reaching the last mile needs to be ensured to combat this public health challenge. It is also high time to conduct operational research to identify the bottle-necks of the IFA supplementation, test and treat, report, and explore strategies that facilitate AMB interventions reaching all children in India.

## Supporting information

S1 Checklist STROBE statement—checklist of items that should be included in reports of observational studies. (DOC)
